# Biomarkers for Assessment of Human Immunodeficiency Virus and its Co-Infection with Hepatitis B and Hepatitis C Viruses: A Comprehensive Review

**DOI:** 10.30699/IJP.2023.1972384.3009

**Published:** 2023-07-15

**Authors:** Mohammad Abdi, Abbas Ahmadi, Aram Mokarizadeh

**Affiliations:** 1 *Department of Clinical Biochemistry, Faculty of Medicine, Kurdistan University of Medical Sciences, Sanandaj, Iran*; 2 *Department of Molecular Medicine, Faculty of Medicine, Kurdistan University of Medical Sciences, Sanandaj, Iran*; 3 *Biopharmaceutical Research Center, AryoGen Pharmed Inc, Alborz University of Medical Sciences, Karaj, Iran*

**Keywords:** Biomarker, Co-infection, HBV, HCV, HIV

## Abstract

Recently, prevalence of hepatitis B virus (HBV), and hepatitis C virus (HCV) co-infection with Human immunodeficiency virus (HIV), has dramatically increased worldwide due to their shared routes of transmission. Compared to the sporadic infection with HIV, HBV, and HCV, concurrent infection with these agents increases the complications of these viruses. Furthermore, co-infection may also alter the therapeutic strategies against HIV. Accordingly, choosing appropriate biomarkers to detect these co-infections is one of the main concerns in the field of diagnostic pathology. Up to now, several markers have been introduced for the simultaneous diagnosis of HIV, HBV, and HCV.

In this regard, serum adenosine deaminase activity (ADA), FibroTests, AST-to-Platelet Ratio Index (APRI), Fibrosis-4, Hyaluronic acid, and micro ribonucleic acids (MiR) have been investigated as potential biomarkers for diagnosis of HIV-HCV/HBV co-infections.

This review summarizes diagnostic values of the current and emerging biomarkers in HIV patients concurrently infected with HBV and HCV.

## Introduction

Acquired immune deficiency syndrome (AIDS) is a potentially life-threatening infection caused by the human immunodeficiency virus (HIV) (1). The worldwide rate of HIV infection by the end of 2021 was about 38.4 million [33.9 million–43.8 million] people and caused more than 500000 [510 000–860 000] death in this year (WHO, 2022) (2, 3). Two important lentiviruses named as human immunodeficiency viruses types 1 and 2 (HIV-1 and HIV-2) have been identified as the main causes of AIDS (4). The HIV-1 mainly spreads by sexual, percutaneous, and perinatal routes (5). Altogether, the HIV-2-infected patients have a lower viral load compared to the HIV-1 individuals, which may explain the lower transmission rates of HIV-2 and the near complete absence of mother-to-infant transmissions. Although HIV-2 infected patients show clinical symptoms like HIV-1, most infected patients do not experience disease progression to AIDS (6, 7). 

Similar to HIV, hepatitis B virus (HBV) and hepatitis C virus (HCV) are among the top 10 leading causes of infectious disease deaths worldwide (8). In fact, due to the shared modes of transmission, dual infections by HIV and HCV/HBV and triple infections are highly prevalent (8-11). Overall, in Western countries it is estimated that 2530% of people living with HIV are coinfected with HCV, and 614% with HBV (8). Furthermore, the prevalence of HBV/HCV is about 1.4% (12).

Due to the significant impact of co-infection on the lifespan, severity of the HIV syndrome, and also the selection of appropriate therapeutic strategies, many attempts have been made to find clinically appropriate biomarkers for diagnosis and screening of HIV and its co-infections with HBV and HCV. 

Given the above introduction, the present review aims to summarize the recent data on the potential diagnostic biomarkers for the HIV co-infection with HBV or HCV. To this end, first, the markers used for the diagnosis of HIV are briefly described. Then, markers used for diagnosis or monitoring of HIV co-infection with HBV and HCV along with indicators used to assess liver damage in co-infected patients are criticized.

**Diagnosis of HIV Mono-Infection**

Detection of HIV specific antigens and antibodies in the biological fluids of infected patients is the main strategy for the diagnosis of HIV-1 infection. Accordingly, many commercially rapid tests are presented as important tools for the surveillance, screening, and diagnosis (13-15).

The main markers for diagnosis of HIV are detecting viral RNA, p24 antigen (a viral core protein), and HIV-specific antibodies (16). On the other hand, viremia and measurement of CD4+ cells are used for the staging purposes. Furthermore, for treatment monitoring, plasma viral load is widely used. While the viral load determines the rate of destruction of the immune system, the number of CD4+ cells reveals the degree of immunodeficiency. Therefore, it is used to assess the stage of infection. Classification of the disease in HIV-infected patients is done according to the CD4+ cell counts and clinical manifestations (e.g. occurrence of the opportunistic infections). In this regard, flow cytometry analysis is the standard method for quantification of CD4+ cells (17).

The first step in the diagnosis of HIV infection is performed by screening antibodies against HIV via enzyme immunoassay (EIA). Additionally, a more efficient nucleic acid amplification test (NAAT) is used for confirmation of the results obtained by EIA. Monitoring tests of HIV-infected individuals includes measurement of CD4+ cell count to evaluate the immune status and viral load to assess the virus replication and changes in the viral genome (18).

According to the last guideline for the diagnosis and treatment of HIV infection presented by the Ministry of Health and Medical Education of Iran, confirmation of the positive cases should be done through an algorithm including three laboratory tests. In the first phase, the immunoassay assessment based on the third or fourth generation of ELISA method or rapid assay is recommended. The second and third assessments should be performed using the fourth generation of ELISA method to confirm the results of the first test. If there are two positive and one negative results, this inconsistency should be confirmed through a nucleic acid amplification test (NAAT). Moreover, updated recommendations advice not to use Western blot (WB) assay in the HIV testing strategies or algorithms. This test takes more time to provide final results and also needs highly skilled staff to perform the test and is costlier. In a recent systematic review study conducted by WHO, two testing strategies including WB/line immunoassays and a combination of HIV rapid diagnostic tests (RDTs) and/ or EIAs were compared. Despite similar accuracy (sensitivity and specificity) found for both strategies, the algorithm used WB/line immunoassays showed more false-negative and indeterminate results. Therefore, resulted in higher number of patients recall and retesting after 14 days. One other important fault of these results was delay in starting the antiretroviral therapy (ART) (19). Basically, WB analysis evaluates the presence of antibodies (IgG) against viral proteins. It has been already proved that WB test fails to identify acute HIV-1 infections. Besides, new generations of immunoassay tests have higher sensitivity compared to the WB, therefore, using the HIV-1 WB for confirmation of these immunoassays can produce false-negative results during seroconversion. More importantly, the use of HIV-1 WB in the previous algorithm misclassifies the majority of HIV-2 infections, which leads to incorrect or delayed diagnosis (20). 

Diagnosis and/or Monitoring of HIV Co-Infection

Early diagnosis of both HIV mono and coinfections with HBV or HCV is essential to effectively manage the infected individuals. Previous guidelines have shown a clear algorithm to determine HIV, HBV and HCV in chronic or acute cases. These include immunoassays and NAAT tests that are accepted by almost all the health organizations worldwide. Table 1 summarizes the tests used for diagnosis of HIV, HBV and HCV and the stage each test is covered. In addition to these approved guidelines, there are some studies that introduced several biochemical markers which directly or indirectly correlate to the HIV mono- or co-infection with HCV and HBV. Accordingly, many attempts have been made to select or find biomarkers with the greatest discriminatory power to distinguish between mono- and co-infected individuals (21-23). Having said this, the following part discusses the most investigated markers that have been proposed in this regard.

**Table 1:** Current laboratory tests use for diagnosis HIV, HBV and HCV in suspected patients.

Disease	Diagnostic test		Disease status		Comment
			Acute	Chronic	
HBV	Immunoassay	HBsAg HBeAg HBcAg	+	+/-	Primary serologic test is HBsAg. Antibodies will reach their peak usually after 6 months. NAAT test remains positive but decreases in chronic state with negative e antigen.
		Anti-HBs Anti-HBe Anti-HBc	- (IgM-HBc positive)	+ (IgG-HBc positive)	
	NAAT		+	+	
HCV	Immunoassay	Anti-HCV Antibody	+	+	If Anti-HCV is negative but clinically indicated, acute HCV is probably indicated and can be evaluated by qualitative NAAT tests. If Anti-HCV is positive, acute state should be confirmed by quantitative NAAT test
	NAAT		+	-	
HIV	Immunoassay	HIV1/2 Antibody	+	+	
	NAAT		+	-	

HBV: Hepatitis B virus; HBsAg: Hepatitis B Surface Antigen; HBcAg: Hepatitis B core Antigen; NAAT: Neucleic acid amplification test; HCV: Hepatitis C virus; HIV: Human immunodeficiency virus.

**Adenosine Deaminase**

Recently, adenosine deaminase (ADA) activity has been introduced as a sensitive and rapid diagnostic marker in evaluation of hepatitis and immunodeficient patients (24-26). 

ADA (EC 3.5.4.4) is a hydrolytic enzyme involved in deamination of adenosine and deoxyadenosine nucleosides, forming inosine and deoxyinosine, respectively (27). This enzyme is widely distributed in human tissues involved in the immune system development (28, 29) through its important role in proliferation and differentiation of the lymphoid cells (30). ADA is also the main regulator of adenosine concentration in plasma, which is involved in the development of inflammatory response and cytokine production (31-33). Two major isoforms of ADA have been isolated with different characteristics. ADA1 exists in all human tissues and ADA2 is the main ADA isoenzyme in the serum (30, 34). Both serum total ADA activity and its isoenzyme activities have frequently been used as diagnostic biomarkers in the screening of infectious diseases (24-26, 35-38). Recently, a significant increase in ADA activity has been reported in HIV–HBV coinfected patients confirming the usefulness of ADA activity as a marker for screening and monitoring HIV-positive and HIVHBV coinfected patients among other diagnostic tools (39).

HIV infection alters serum ADA activity, which has an indirect correlation with the reduction of CD4+ cell counts (38). It is well established that co-infection of HIV with HBV increases the ADA activity much more than the mono-infection with HIV or HBV (33, 37-39). Therefore, since the increase in the enzyme activity occurs in the early stages of the disease process, some studies have suggested that elevated ADA activity should be considered as a potent marker for the detection of HIV-HBV co-infection (39). More involvement of monocytemacrophage system in the HIV co-infection compared to the mono-infection is the main cause of higher ADA activity in co-infected patients since this system participates in the immune responses and regulates the exact amounts of adenosine in the immune cells. Additionally, it has been shown that ADA activity has a negative correlation with CD4+ cell counts indicating that serum ADA activity is increased whilstCD4+ cell count is decreased during HIV progression. On the other hand, a strong direct correlation has been found between total ADA and ADA2 activities. These results suggest that serum ADA can be considered as a sensitive marker of an ongoing biological insult to the host immune system (25, 37, 38). 

The co-enhancement in the rise of total ADA and ADA2 activities can be explained by the fact that the monocytemacrophage system, which is involved in the immune responses and regulates the exact amounts of adenosine in the immune cells, is the main source of ADA2 isoenzyme (36). In fact, the increase in total ADA activity might be due to the increase in ADA2 isoenzyme activity, as a strong direct correlation (R2=0.945, *P*<0.05) has been observed between total ADA and ADA2 activities (38, 39). 

The clinical sensitivity and specificity of the serum total ADA and ADA2 activities for the differentiation of the HIV-infected patients from healthy ones are about 90.9%, 90.27%; and 93%, 90%, respectively, as compared to the EIA and NAAT results (37). A recent study has evaluated the sensitivity and specificity of these enzymes in differentiating HIV mono- and co-infections with HBV and HCV in the early stages of disease process. Therefore, cut off values for the total ADA, ADA1, and ADA2 enzyme activities were scored at 58.33 U/L, 4.95 U/L, and 47.73 U/L, respectively. This study also showed an acceptable sensitivity, specificity, and significant area under curve for both total ADA and ADA2 enzyme activities (Table 2) (39). 

Previous studies suggested that determination of the serum total ADA (or ADA2) activity may be used as an alternative for the CD4+ cell count, HIV antibody screening test by Enzyme-linked immunosorbent assay (ELISA) (37), and viral load determination. Finally, it should be noted that determination of the total ADA or ADA2 isoenzyme activities is a simple, rapid, and inexpensive diagnostic tool for screening and monitoring of HIV-infected patients and HIV-HBV co-infection, compared to the current costly, laborious, and timeconsuming markers such as CD4+ cell count and viral load determination. Hence, it might be of great value for immediate diagnosis of the disease with a positive impact on the patient outcome. 

**Table 2:** Diagnostic values for total ADA and its ADA2 isoenzyme to differentiate HIV mono-infection from HBV and HCV co-infection patients

	Sensitivity (%)	Specificity (%)	PPV	NPV	AUC
Total ADA (U/L)	65	82	0.87	0.56	0.732
ADA2 (U/L)	72	76	0.84	0.60	0.714

AUC: area under curve; NPV: negative predictive value; PPV: positive predictive value; U/L: units per liter of enzyme activity.

**Liver Enzymes**

Elevated levels of the liver enzymes such as alanine aminotransferase (ALT), aspartate aminotransferase (AST), and alkaline phosphatase (ALP) have been clearly shown in HIV-HBV, HIV-HCV, and HIV-HBV-HCV co-infected individuals (40-42). Increased level of the liver enzymes, especially AST and ALT, can be proposed as a predictive marker for the possible liver injuries by the viral hepatitis in the patients with HIV infection. However, different studies have reported different values for the enzyme activity elevation and most of them have not found any significant differences in this regard despite the trend to elevate in enzyme activity (43-45). Discrepancy between liver enzyme levels in different studies may be due to the difference in study designs, duration of the viral hepatitis infection, and patient’s selection (i.e., existence of the patients with chronic alcoholism or other drug induced hepatotoxicity in the population) (46). In addition, HIV can cause liver fibrosis development and elevate liver enzyme levels by infecting the hepatic or kupffer cells (47). Furthermore, the degree of the liver injury may be high in co-infection with HBV and HCV. Although the rise in transaminases and ALP has been shown in almost all studies, most of these researches insist only on transaminases. Simply, the higher ALP activity in the HIV-HBV co-infected patients is not significant; therefore, it cannot be used as a marker for the diagnosis of liver involvement in the HIV-infected patients. However, the AST and ALT activities show higher and significant serum level in the co-infected patients, therefore, they can be considered as simple and accessible markers for the early diagnosis of liver injuries in the HIV patients (possibly due to co-infection of HIV with HCV or HBV). Although transeaminases are sensitive markers for the early detection of liver injuries, they cannot discriminate which type of viral hepatitis (HBV or HCV) is co-infected with HIV. Furthermore, it seems that the elevation of AST activity is much more than that of ALT. For instance, in a recent study, co-infected patients had higher proportions of the elevated values of AST (86.6%) compared to that of ALT (56.7%) (48). It seems that aminotransferases have high association with histological activity, but the reported positive predictive value (PPV) (about 60%) for these markers is low. Conversely, their high negative predictive value (NPV) (about 96%) makes these tests effective biomarkers for excluding the moderate histological activity in the patients with normal values. However, there are some studies that reject these results (40, 49). It is illustrated that sampling, histological classification, patient’s population, and the analyzing method may affect the test results. Even though these tests are very practical, it should not be ignored that the increase in the hepatic enzymes can also be caused by other reasons such as alcoholism, lipid lowering drugs, antibiotics, and direct hepatic damage due to HIV (46).

**CD4 Cell Count**

Measurement of the CD4+ cell is a good approach for monitoring disease activity and/or treatment response in the HIV-positive patients. Moreover, CD4+ cell count has been introduced as a marker for detection of HIV co-infection with HBV and HCV (38). It has been shown that co-infection with HBV or HCV or with both of them can strongly decrease the CD4+ count, and the decline in CD4+ count is significantly higher in the triple co-infection (HIV/HBV/HCV) than in double co-infection (HIV/HBV or HIV/HCV) (38, 44, 50). This low CD4+ count in the HIV co-infected patients may be related to the increase in HIV, HBV, and HCV reproduction reflecting an immune-suppressed condition (43). In a study conducted to discriminate HIV mono-infection from HIV-HCV and HIV-HBV co-infections, the cut-off value of 846 cells/μl was set; however, this value was lower in other studies (43, 45, 50). On the other hand, it is shown that CD4+ cell count cannot differentiate the type of co-infection (HBV or HCV), though the decrease in CD4+ cell count could be suggested as a predicting marker for possible liver involvement (51-53). Furthermore, it should be mentioned that the main reason for reduction in the CD4+ cell is the progress in the HIV disease, and the reduction in the CD4+ cell count due to viral hepatitis may only be proposed when this decrease occurs dramatically and in the first stages of HIV infection.

**Determination of Liver Fibrosis**

There have been attempts to develop and validate some non-invasive tests for staging the hepatic fibrosis as surrogate markers for the liver biopsy. Three indirect blood fibrosis markers, AST-to-platelet ratio index (APRI), the Fibrosis-4 score (FIB-4), and FibroTest were included in the 2014 WHO guidelines for management of HCV in low-income countries (54, 55). These tests have been validated in HIV, HBV, and HCV mono-infections as well as HIV-HBV and HIV-HCV co-infections in the different racial and geographical areas such as North America, Asia, and Europe (56-60).

**The FibroTest: **FibroTest (also known as Fibrosure in the US) is an index for the evaluation of liver scarring (40, 61-63). FibroTest score can be calculated by measuring five serum markers including alfa2-macroglobulin (α2M) [g/l], apolipoproteinA1 (Apo A1) [g/l], total bilirubin (tBil) [micromoles/l], haptoglobin (Hp) [g/l], and Gamma-glutamyltranspeptidase (GGT) [IU/l]. The above values are located in the following formula:

z=4.467×log[α2M]-1.357×log[Hp]+1.017×log[GGT]+0.0281×[Age (years)]+1.737×log[tBil]-1.184×[Apo A1]+0.301 Sex [Female=0,Male=1]-5.54

As it can be concluded from the above formula, FibroTest is not affected by the ethnicity, viral load, and the presence of co-morbidities. However, in the maximum of 5% of cases such as acute hepatitis and extrahepatic cholestasis, the test has no applicability.

**Fig. 1:**
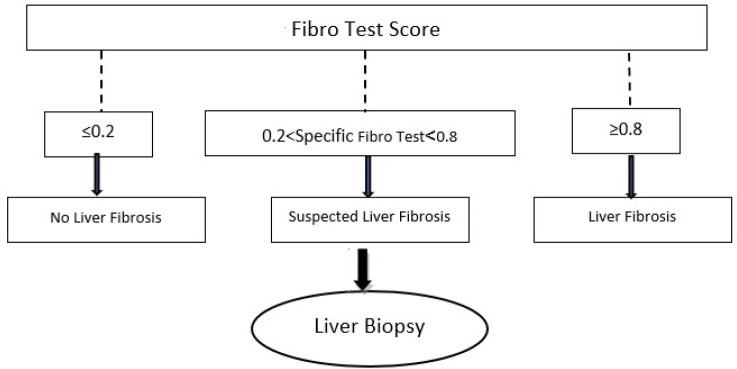
Schematic representation of diagnosis of liver fibrosis according to the FibroTest score.

In addition to FibroTest, Actitest is another index that has been used for measuring the liver inflammation; it uses alanine aminotransferase (ALT) as well as the above five markers. The FibroTest and Actitest are non-invasive, accurate, and highly reproducible indices that simply can score liver damage by evaluating the serum biochemical markers (40, 41, 61-63). Previous studies have illustrated their potential usefulness in predicting the severity of necro-inflammation and fibrosis. Furthermore, Actitest is more sensitive than FibroTest, hence, Actitest can be used as a screening test in the evaluation of hepatic involvement. In the severe liver injuries, conditions that occur in HDV co-infection, GGT is introduced as an accurate marker that can be used in HBV-related inflammation (40). In addition, FibroTest has high NPV (92%) for the diagnosis of fibrosis and a value of ≤0.20, which can reliably exclude significant fibrosis. On the other hand, FibroTest scores higher than 0.80 with PPV=92% can accurately diagnose F2-F4 fibrosis scores (i.e. >0.20 and <0.80) that may obviate biopsies (Figure 1).

In the patients with advanced fibrosis caused by HBV, α2M will highly increase. On the other hand, in this stage, apolipoprotein A1 and haptoglobin are the main decreasing biomarkers. However, α2-macroglobulin has also a positive correlation with alcoholic-related liver fibrosis as well as chronic HCV (40, 62). Protease inhibitor activity of α2M increases production of this protein in the hepatic stellate cells, resulting in enhanced fibrosis through reducing the destruction of extracellular matrix proteins (64). Similar to HBV-related fibrosis, Apo A1 is also reduced in alcoholic liver disease and chronic HCV (40).Considering the difference between the pathogenesis of alcoholic liver disease and chronic hepatitis B and C, these similarities suggest the potential applicability of these proteins in other fibrotic conditions. As a result, attention should be paid when using this index since it is not specifically used for diagnosis of viral-related fibrosis, and it may be altered in alcoholic-induced fibrosis (65).

Overall, the FibroTest is useful for identification of HBV-related fibrosis, and the Actitest seems useful for excluding significant necro-inflammation.

**FIB-4**

Similar to FibroTest, Fibrosis-4 (FIB-4) is a liver scarring index. This test produces interesting results in the patients co-infected with HIV and HCV using the following formula (66):

FIB-4=(Age (years)×AST(U/L))/(Platelets (〖10〗^9/L)×√(ALT(U/L)))

**Fig. 2:**
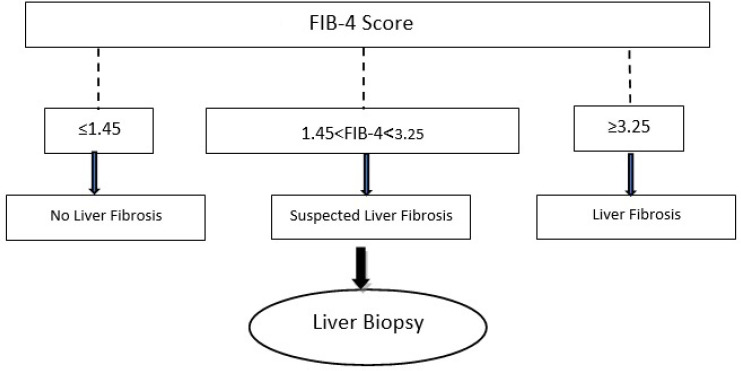
Schematic representation of diagnosis of liver fibrosis according to the FIB-4 score.

The FIB-4 index has an area under the receiver operating characteristic (ROC) curve of 0.76. A threshold value of <1.45 has a negative predictive value for the exclusion of extended fibrosis of 90%. However, a threshold value of >3.25 has a positive predictive value for the diagnosis of extended fibrosis of 65% (Figure 2) (66).

The FIB-4 index has some valuable advantages. Firstly, its major benefit is that the FIB-4 index is very applicable and calculations are simple and quick. Secondly, physicians can instantly measure the index in clinic. Finally, it is really cheap. In fact, the method for obtaining the FIB-4 index does not require especial device and additional costs to calculate this index, since the constitutive FIB-4 data are included in the standard examination of the liver function (age, AST, ALT, platelet count). These facts make FIB-4 index a better biomarker than the current evaluated non-invasive markers for the liver examination in the patients with viral hepatitis, particularly in low-income countries with high rates of HBV and its co-infections with HCV and HIV. The accuracy of this marker is to the extent that it can differentiate about 75% of the liver involvement; this is the amount that can be detected by FibroTest (67-69).

FIB-4 has the area under curve (AUC) of about 85% for the diagnosis of sever fibrosis (grade F3 and above). In particular, the FIB-4 index can be used for differentiating high-grade from moderate-grade fibrosis. Overall, the results obtained by this non-invasive indicator are consistent with 70% of biopsies (70).

Ninety-two percent of the calculated results by FIB-4 index lower than 1.45 is consistent with the degree of liver fibrosis. On the other hand, FIB-4 index >3.25 has 76% agreement with the patient illness. In low value cases of FIB-4 index, the discordance is related to the young age and normal platelet count. For a FIB-4 index >3.25, old age and low platelet count explain the FIB-4 failures. The FIB-4 index is more practical than FibroTest because, contrary to FibroTest, it does not require standardization of the biochemical values to be used in a reliable manner (70).

**The AST-to-Platelet Ratio Index (APRI)**

APRI is the most reasonably-priced laboratory marker for measuring liver fibrosis. In addition, especially because most of the HBV- and HCV-infected patients live in the regions with limited healthcare resources (71, 72), this test is the most affordable tool in these low-income countries for evaluation of the early liver damage in the HIV mono-infected and co-infected patients with viral hepatitis. Although APRI does not have a high prognostic value (AUC=0.76), it is a strong tool for excluding the significant fibrosis (73). An APRI score ≤0.5 is the preferred cut-off value for excluding the significant fibrosis in average frequency areas. By using this cut-off, about 33% of the liver biopsies can be avoided. However, because of low PPV, high APRI values (>1.5) are not sufficient index for classification of liver fibrosis in high prevalence regions (74). The second most important use of APRI is application of this marker in ruling out the patients without cirrhosis. In the values equal to 1 or 2, APRI has 91% and 94% NPV, respectively for excluding cirrhosis. On the contrary, due to low PPV, it cannot be used for including liver cirrhosis. The interesting part is that, among fibrosis markers, there is no significant difference between the AUCs for the significant fibrosis of the APRI (0.76), FibroTest (0.79), FibroMeter (0.78), and HepaScore (0.76) (75). Previously, it was supposed that due to HIV-related or antiretroviral-related thrombocytopenia, the accuracy of APRI may decrease; however, surprisingly the accuracy of the test does not diminish in the co-infected patients (73, 76).

Besides, it seems that APRI values strictly depend on the CD4+ counts. There are some investigations that prove the effect of CD4+ counts on the APRI performance in the HIV-HCV co-infected patients (77-79). The results of ROC curve analysis of these studies show that the AUC obviously decreased in the co-infected patients with higher CD4+ counts compared to the patients with lower CD4+ counts (≤250 cells/mm3) (80). In those with higher CD4+ cell counts, the AUC is similar to that found in HIV-negative patients (81). Decline in the CD4+ counts directly affects the strength of APRI in classifying liver fibrosis. It is shown that APRI properly grades liver fibrosis in mono- and co-infected patients with higher CD4+ counts, but only a low proportion of patients with low CD4+ count is clearly classified with this index. In addition, although liver biopsy in both mono- and co-infected patients with low CD4+ is unavoidable, determination of the liver injury in both mono- and co-infected patients with higher CD4+ count is much less required for this operation. APRI < 0.5 has high negative predictive value to exclude significant fibrosis (82). On the other hand, it has been proved that in the patients with low CD4+ count, this NPV significantly decreased suggesting that APRI has more limited utility in this subgroup when compared to those with higher CD4+ counts and HIV-negative patients (73). Low diagnostic value of APRI in the patients with low CD4+ count can be explained by this fact that HIV infection can cause thrombocytopenia. Furthermore, HAART may develop hepatotoxicity by mitochondrial toxicity or metabolic dysfunction, and these processes can contribute to increased AST levels. Finally, treating these patients with antiretroviral agents may progress the liver fibrosis through steatosis; besides, non-invasive markers cannot reflect this change that was validated in groups of patients with less coexisting risk factors for disease severity (80).

**Hyaluronic Acid (HA)**

Hyaluronic acid is one of the most important glycosaminoglycans (GAGs), which is widely distributed throughout the connective, epithelial, and neural tissues (83, 84). Recently, hyaluronic acid has been proposed as a marker for diagnosing and differentiating the advanced stages of fibrosis (85). Hyaluronic acid has a high NPV (about 100%) in values lower than 60 µg/L, and it can well bring out patients without high stages of liver damage (cirrhosis). However, it seems that PPV for this marker is not strong enough for diagnosing the end-stage liver fibrosis (58). The increased serum hyaluronic acid level may be the consequence of an increase in the hepatic stellate cell production along with a decrease in elimination by hepatic sinusoidal endothelial cells. Previous results clearly proved a correlation between serum concentration of hyaluronic acid and liver fibrosis in the patients with HBV and HCV co-infected with HIV (86-91). 

It has been shown that the combination of hyaluronic acid with albumin and AST has more discriminating value than single hyaluronic acid (58). When used together, these biomarkers may act as a surrogate for the liver biopsy and can truly differentiate patients without high-grade liver fibrosis. Overall, hyaluronic acid, solely or in combination with AST and albumin, can be used for diagnosing liver fibrosis in the patients with high risk of liver injuries such as HIV and HBV/HCV co-infections (92). The results of the previous studies did not show any deviation from the results obtained by this marker in the HCV or HBV co-infected and mono-infected patients, hence it can be concluded that co-infection of HBV with HCV or HIV has no effect on the test results. Although the optimal AUC for this marker is approximately 0.878, similar to FibroTest, APRI, and FIB-4, hyaluronic acid appears to be non-specific and is considered more as a fibrosis marker rather than being a disease specific index (58).

**Micro RNAs (miRs)**

MicroRNAs (miRs) are non-coding, conserved, and small (18-22 nucleotides long) ribonucleic acids that regulate gene expression by targeting 3ʹ end of the messenger RNA (mRNA) (93, 94). Therefore, they can involve in many cellular processes such as differentiation, cell proliferation, and apoptosis. Recently, the effect of these factors on viral infection, fibrosis, and hepatocellular carcinoma (HCC) has been demonstrated. In addition, some of these factors have been proposed as markers of the liver injury (95, 96). 

It has been shown that the level of miR expression in the chronic HCV patients can determine the stage of the disease or predict the response to the treatment; hence, they can be used as diagnostic markers (97, 98). For instance, the increase in miR-21 in HCV individuals results in the increase in the viral load, liver fibrosis, and tumorigenesis (99, 100). The use of these markers in the evaluation of responses to therapy can also be useful; for instance, tumors with low level of miR-26 show better response to the treatment with IFN than tissues with high expression of this miR (101). MiR-122 has a main role in the HCV replication and reduction of miR-122 level in the liver which leads to the reduction in HCV viral load (102, 103). Furthermore, increased expression of miR-122 in the patients with HBV will be the outcome of severe liver injury (104, 105). In HIV-1, the infected T cell expression of miR-122 also increases, and interestingly the miR-122 in non-infected cells is undetectable (106-108). On the other hand, increased expression of some certain miRs such as miR-29, results in a decrease in HCV replication (109). MiR-29 can weaken the HIV-1 genome by targeting 3′-untranslated region (UTR) (110). The reduced level of this miR in the patients with HBV is proposed as a prognostic marker of fibrosis (103). By targeting 3′-UTR of HIV-1 genome and inhibiting this region, miR-223 could act as an anti-HIV miR (111). Furthermore, it has been shown that miR-223 may be considered as a biomarker in the patients with HBV-related hepatocellular carcinoma (HCC) (112). Nonetheless, conflicting results in this regard are abundant. For example, Witwer et al., (113) investigated the correlation between miRs profile and CD4+ cell count and viral load in the HIV patients and healthy subjects where there was no relationship between miR profile and CD4+ cell count. Conversely, in another study, this finding was rejected and it was shown that miR profile strictly correlates with the grade and progression of HIV. Recently, miR-122, miR-22, and miR-24a are proposed as markers of liver injury in the HIV co-infected patients with viral hepatitis (114). These markers were highly up-regulated in the HIV co-infected patients compared to the HIV mono-infected ones and had a high correlation with APRI score and FIB-4 index. Although circulating levels of miR-122 and miR-34a are higher in the HIV-HCV co-infected patients, the increasing miR in HIV-HBV co-infected subjects is miR-22 (114).

## Conclusion

In HIV-infected individuals, co-infection with HBV and/or HCV is common due to shared modes of transmission. Several markers have been proposed for the follow-up of the disease in HIV, HBV, and HCV co-infected patients. It is known that HIV accelerates progression of the liver disease and results in increased morbidity and mortality associated with the viral hepatitis. One of the good indices for evaluating the liver injury in HIV-viral hepatitis co-infection is Fibrotest. Despite the high diagnostic value of FibroTest, there is no significant difference between the diagnostic value of this index and that of the other indices such as FIB-4 and APRI. Hence, the APRI score is suggested for monitoring the disease in the co-infected patients, especially in low-income regions. Furthermore, it should not be ignored that there is no significant difference between the prognostic value of these indices in diagnosing mono-infection and co-infection. Finally, because none of these markers can totally determine liver fibrosis, increasing attention to the novel markers like miRs seems necessary. Besides, one important issue that must be pointed is that due to the lower diagnostic value, these markers are not able to completely differentiate co-infection diseases from the other non-infection inflammatory situations. Therefore, considering clinical history should be remained as a main step in management of HIV patients with viral co-infection(s).

## Funding

 None.

## Conflict of Interest

The authors report no conflicts of interest. This article is the outcome of an in-house financially non-supported study.

## References

[1] Pham QD, Wilson DP, Law MG, Kelleher AD, Zhang L (2014). Global burden of transmitted HIV drug resistance and HIV-exposure categories: a systematic review and meta-analysis. AIDS..

[2] Stabinski L, O'Connor S, Barnhart M, Kahn RJ, Hamm TE (2015). Prevalence of HIV and hepatitis B virus co-infection in sub-Saharan Africa and the potential impact and program feasibility of hepatitis B surface antigen screening in resource-limited settings. J Acquir Immune Defic Syndr..

[3] Fettig J, Swaminathan M, Murrill CS, Kaplan JE (2014). Global epidemiology of HIV. Infect Dis Clin North Am..

[4] Seitz R (2016). Human Immunodeficiency Virus (HIV). Transfusion medicine and hemotherapy : offizielles Organ der Deutschen Gesellschaft fur Transfus Immunhamatol..

[5] Hladik F, McElrath MJ (2008). Setting the stage: host invasion by HIV. Nat Rev Immunol..

[6] Sharp PM, Hahn BH (2011). Origins of HIV and the AIDS pandemic. Cold Spring Harb Perspect Med..

[7] Bartolo I, Rocha C, Bartolomeu J, Gama A, Marcelino R, Fonseca M (2009). Highly divergent subtypes and new recombinant forms prevail in the HIV/AIDS epidemic in Angola: new insights into the origins of the AIDS pandemic. Infect Genet Evol..

[8] Alter MJ (2006). Epidemiology of viral hepatitis and HIV co-infection. Journal of hepatology..

[9] Diop-Ndiaye H, Toure-Kane C, Etard JF, Lo G, Diaw P, Ngom-Gueye NF (2008). Hepatitis B, C seroprevalence and delta viruses in HIV-1 Senegalese patients at HAART initiation (retrospective study). J Med Virol..

[10] Hosseini M, SeyedAlinaghi S, Kheirandish P, Esmaeli Javid G, Shirzad H, Karami N (2010). Prevalence and correlates of co-infection with human immunodeficiency virus and hepatitis C virus in male injection drug users in Iran. Arch Iran Med..

[11] Moradi A, Khodabakhshi B, Sadeghipour M, Besharat S, Tabarraei A (2011). Concurrent infections of hepatitis C and HIV in hepatitis B patients in the north-east of Iran. Tropic Doctor..

[12] Tyson GL, Kramer JR, Duan Z, Davila JA, Richardson PA, El-Serag HB (2013). Prevalence and predictors of hepatitis B virus coinfection in a United States cohort of hepatitis C virus-infected patients. Hepatology (Baltimore, Md..

[13] Agutu CA, Ngetsa CJ, Price MA, Rinke de Wit TF, Omosa-Manyonyi G, Sanders EJ (2019). Systematic review of the performance and clinical utility of point of care HIV-1 RNA testing for diagnosis and care. PLoS One..

[14] Marinho FLO, Santos NLL, Neves SPF, Vasconcellos LS (2020). Performance evaluation of eight rapid tests to detect HIV infection: A comparative study from Brazil. PLoS One..

[15] Mourez T, Lemee V, Delbos V, Delaugerre C, Alessandri-Gradt E, Etienne M (2018). HIV rapid screening tests and self-tests: Be aware of differences in performance and cautious of vendors. EBioMedicine..

[16] Guarner J (2017). Human immunodeficiency virus: Diagnostic approach. Semin Diagn Pathol..

[17] Simon V, Ho DD, Abdool Karim Q (2006). HIV/AIDS epidemiology, pathogenesis, prevention, and treatment. Lancet..

[18] Grossman Z, Kra-Oz Z (2013). [HIV testing: the medical laboratory role in HIV diagnosis and monitoring]. Harefuah..

[19] World Health O (2019 ). WHO recommends countries move away from the use of western blotting and line immunoassays in HIV testing strategies and algorithms: policy brief.

[20] Branson BM, Owen SM, Wesolowski LG, Bennett B, Werner BG, Wroblewski KE (2014). Laboratory testing for the diagnosis of HIV infection : updated recommendations. Centers for Disease C Prevention Association of Public Health L National Center for Hiv/Aids VH Prevention TBPDoHA.

[21] Nunez M, Puoti M, Camino N, Soriano V (2003). Treatment of chronic hepatitis B in the human immunodeficiency virus-infected patient: present and future. Clin Infect Dis..

[22] Schiavini M, Angeli E, Mainini A, Uberti-Foppa C, Zerbi P, Sagnelli C (2011). Fibrosis progression in paired liver biopsies from HIV/HCV co-infected patients. Hepatitis Monthly..

[23] Thio CL, Seaberg EC, Skolasky R Jr, Phair J, Visscher B, Munoz A (2002). HIV-1, hepatitis B virus, and risk of liver-related mortality in the Multicenter Cohort Study (MACS). Lancet..

[24] Niedzwicki JG, Kouttab NM, Mayer KH, Carpenter CC, Parks RE Jr, Abushanab E (1991). Plasma adenosine deaminase2: a marker for human immunodeficiency virus infection. J Acquir Immune Defic Syndr (1988)..

[25] Chittiprol S, P S, S BR, R RG, V SS, K SD (2007). Plasma adenosine deaminase activity among HIV1 Clade C seropositives: Relation to CD4 T cell population and antiretroviral therapy. Clin Chim Act..

[26] Kaya S, Cetin ES, Aridogan BC, Arikan S, Demirci M (2007). Adenosine deaminase activity in serum of patients with hepatitis -- a useful tool in monitoring clinical status. J Microbiol Immunol Infect..

[27] Phillis J (1991). Adenosine and adenine nuleotides as regulators of cellular function.

[28] Conway EJ, Cooke R (1939). The deaminases of adenosine and adenylic acid in blood and tissues. The Biochemical journal..

[29] Van der Weyden MB, Kelley WN (1976). Human adenosine deaminase. Distribution and properties. J Biol Chem..

[30] Gakis C (1996). Adenosine deaminase (ADA) isoenzymes ADA1 and ADA2: diagnostic and biological role. Eur Respir J..

[31] Zidek Z (1999). Adenosine - cyclic AMP pathways and cytokine expression. Eur Cytokine Netw..

[32] Goodarzi MT, Abdi M, Tavilani H, Nadi E, Rashidi M (2010). Adenosine deaminase activity in COPD patients and healthy subjects. Iranian journal of allergy, asthma, and immunology..

[33] Khodadadi I, Vahedi MS, Abdi M, Daneshkhah N, Rahbari R, Menbari S (2014). Evaluation of adenosine deaminase (ADA) isoenzymes activity and tumor necrosis factor-alpha (TNFalpha) concentration in chronic heart failure. EXCLI J..

[34] Ungerer JP, Oosthuizen HM, Bissbort SH, Vermaak WJ (1992). Serum adenosine deaminase: isoenzymes and diagnostic application. Clin Chem..

[35] Kalkan A, Bulut V, Erel O, Avci S, Bingol NK (1999). Adenosine deaminase and guanosine deaminase activities in sera of patients with viral hepatitis. Memorias Institut Oswaldo Cruz..

[36] Tsuboi I, Sagawa K, Shichijo S, Yokoyama MM, Ou DW, Wiederhold MD (1995). Adenosine deaminase isoenzyme levels in patients with human T-cell lymphotropic virus type 1 and human immunodeficiency virus type 1 infections. Clin Diagn Lab Immunol..

[37] Abdi M, Ahmadi A, Roshany D, Khodadadi I, Javid S, Shahmohammad-Nezhad S (2013). Diagnostic value of serum adenosine deaminase activity in HIV infected patients of Kurdish population. Clin Lab..

[38] Khodadadi I, Abdi M, Ahmadi A, Wahedi MS, Menbari S, Lahoorpour F (2011). Analysis of serum adenosine deaminase (ADA) and ADA1 and ADA2 isoenzyme activities in HIV positive and HIV-HBV co-infected patients. Clin Biochem..

[39] Abdi M, Rahbari R, Khatooni Z, Naseri N, Najafi A, Khodadadi I (2016). Serum Adenosine Deaminase (ADA) Activity: A Novel Screening Test to Differentiate HIV Monoinfection From HIV-HBV and HIV-HCV Coinfections. J Clin Lab Anal..

[40] Myers RP, Tainturier MH, Ratziu V, Piton A, Thibault V, Imbert-Bismut F (2003). Prediction of liver histological lesions with biochemical markers in patients with chronic hepatitis B. J Hepatol..

[41] Myers RP, Benhamou Y, Imbert-Bismut F, Thibault V, Bochet M, Charlotte F (2003). Serum biochemical markers accurately predict liver fibrosis in HIV and hepatitis C virus co-infected patients. AIDS..

[42] Vermehren J, Vermehren A, Mueller A, Carlebach A, Lutz T, Gute P (2012). Assessment of liver fibrosis and associated risk factors in HIV-infected individuals using transient elastography and serum biomarkers. BMC Gastroenterol..

[43] Wondimeneh Y, Alem M, Asfaw F, Belyhun Y (2013). HBV and HCV seroprevalence and their correlation with CD4 cells and liver enzymes among HIV positive individuals at University of Gondar Teaching Hospital, Northwest Ethiopia. Virol J..

[44] Lodenyo H, Schoub B, Ally R, Kairu S, Segal I (2000). Hepatitis B and C virus infections and liver function in AIDS patients at Chris Hani Baragwanath Hospital, Johannesburg. East Afr Med J..

[45] Tripathi AK, Khanna M, Gupta N, Chandra M (2007). Low prevalence of hepatitis B virus and hepatitis C virus co-infection in patients with human immunodeficiency virus in Northern India. J Assoc Physicians India..

[46] Mata-Marin JA, Gaytan-Martinez J, Grados-Chavarria BH, Fuentes-Allen JL, Arroyo-Anduiza CI, Alfaro-Mejia A (2009). Correlation between HIV viral load and aminotransferases as liver damage markers in HIV infected naive patients: a concordance cross-sectional study. Virol J..

[47] Cao YZ, Dieterich D, Thomas PA, Huang YX, Mirabile M, Ho DD (1992). Identification and quantitation of HIV-1 in the liver of patients with AIDS. AIDS..

[48] Hooja S, Singhal A, Bachhiwal R, Yadav R, Vyas N (2015). Hepatitis B virus seroprevalence and its correlation with CD4 cells and liver enzymes among human immunodeficiency virus positive individuals at a tertiary care hospital in North-West India. Int J Appl Basic Med Res..

[49] ter Borg F, ten Kate FJ, Cuypers HT, Leentvaar-Kuijpers A, Oosting J, Wertheim-van Dillen PM (1998). Relation between laboratory test results and histological hepatitis activity in individuals positive for hepatitis B surface antigen and antibodies to hepatitis B e antigen. Lancet..

[50] Adewole OO, Anteyi E, Ajuwon Z, Wada I, Elegba F, Ahmed P (2009). Hepatitis B and C virus co-infection in Nigerian patients with HIV infection. J Infect Dev Ctries..

[51] Leierer G, Rieger A, Schmied B, Sarcletti M, Ollinger A, Wallner E (2021). A Lower CD4 Count Predicts Most Causes of Death except Cardiovascular Deaths The Austrian HIV Cohort Study. Int J Environ Res Public Health..

[52] Milic J, Menozzi V, Schepis F, Malagoli A, Besutti G, Franconi I (2020). Liver steatosis and nonalcoholic fatty liver disease with fibrosis are predictors of frailty in people living with HIV. AIDS..

[53] Perazzo H, Cardoso SW, Yanavich C, Nunes EP, Morata M, Gorni N (2018). Predictive factors associated with liver fibrosis and steatosis by transient elastography in patients with HIV mono-infection under long-term combined antiretroviral therapy. J Int AIDS Soc..

[54] Organization WH (2014). Guidelines for the screening, care and treatment of persons with hepatitis C infection.

[55] Wai CT, Greenson JK, Fontana RJ, Kalbfleisch JD, Marrero JA, Conjeevaram HS (2003). A simple noninvasive index can predict both significant fibrosis and cirrhosis in patients with chronic hepatitis C. Hepatology (Baltimore, Md..

[56] DallaPiazza M, Amorosa VK, Localio R, Kostman JR, Lo Re V, 3rd (2010). Prevalence and risk factors for significant liver fibrosis among HIV-monoinfected patients. BMC Infect Dis..

[57] Kelleher TB, Mehta SH, Bhaskar R, Sulkowski M, Astemborski J, Thomas DL (2005). Prediction of hepatic fibrosis in HIV/HCV co-infected patients using serum fibrosis markers: the SHASTA index. J Hepatol..

[58] Moodie EE, Pant Pai N, Klein MB (2009). Is antiretroviral therapy causing long-term liver damage? A comparative analysis of HIV-mono-infected and HIV/hepatitis C co-infected cohorts. PLoS One..

[59] Mohamadnejad M, Montazeri G, Fazlollahi A, Zamani F, Nasiri J, Nobakht H (2006). Noninvasive markers of liver fibrosis and inflammation in chronic hepatitis B-virus related liver disease. Am J Gastroenterol..

[60] Poynard T, Zoulim F, Ratziu V, Degos F, Imbert-Bismut F, Deny P (2005). Longitudinal assessment of histology surrogate markers (FibroTest-ActiTest) during lamivudine therapy in patients with chronic hepatitis B infection. Am J Gastroenterol..

[61] Imbert-Bismut F, Ratziu V, Pieroni L, Charlotte F, Benhamou Y, Poynard T (2001). Biochemical markers of liver fibrosis in patients with hepatitis C virus infection: a prospective study. Lancet..

[62] Poynard T, Imbert-Bismut F, Munteanu M, Messous D, Myers RP, Thabut D (2004). Overview of the diagnostic value of biochemical markers of liver fibrosis (FibroTest, HCV FibroSure) and necrosis (ActiTest) in patients with chronic hepatitis C. Comp Hepatol..

[63] Kawser CA, Iredale JP, Winwood PJ, Arthur MJ (1998). Rat hepatic stellate cell expression of alpha2-macroglobulin is a feature of cellular activation: implications for matrix remodelling in hepatic fibrosis. Clin Sci (Lond)..

[64] Sumida Y, Nakajima A, Itoh Y (2014). Limitations of liver biopsy and non-invasive diagnostic tests for the diagnosis of nonalcoholic fatty liver disease/nonalcoholic steatohepatitis. World J Gastroenterol..

[65] Sterling RK, Lissen E, Clumeck N, Sola R, Correa MC, Montaner J (2006). Development of a simple noninvasive index to predict significant fibrosis in patients with HIV/HCV coinfection. Hepatology (Baltimore, Md..

[66] Castera L, Vergniol J, Foucher J, Le Bail B, Chanteloup E, Haaser M (2005). Prospective comparison of transient elastography, Fibrotest, APRI, and liver biopsy for the assessment of fibrosis in chronic hepatitis C. Gastroenterology..

[67] Resino S, Sanchez-Conde M, Berenguer J (2012). Coinfection by human immunodeficiency virus and hepatitis C virus: noninvasive assessment and staging of fibrosis. Curr Opin Infect Dis..

[68] Xu XY, Kong H, Song RX, Zhai YH, Wu XF, Ai WS (2014). The effectiveness of noninvasive biomarkers to predict hepatitis B-related significant fibrosis and cirrhosis: a systematic review and meta-analysis of diagnostic test accuracy. PLoS One..

[69] Vallet-Pichard A, Mallet V, Nalpas B, Verkarre V, Nalpas A, Dhalluin-Venier V (2007). FIB-4: an inexpensive and accurate marker of fibrosis in HCV infection comparison with liver biopsy and fibrotest. Hepatology (Baltimore, Md..

[70] Gower E, Estes C, Blach S, Razavi-Shearer K, Razavi H (2014). Global epidemiology and genotype distribution of the hepatitis C virus infection. Journal of hepatology..

[71] Sunbul M (2014). Hepatitis B virus genotypes: global distribution and clinical importance. World J Gastroenterol..

[72] Shaheen AA, Myers RP (2007). Diagnostic accuracy of the aspartate aminotransferase-to-platelet ratio index for the prediction of hepatitis C-related fibrosis: a systematic review. Hepatology (Baltimore, Md..

[73] Vinikoor MJ, Sinkala E, Mweemba A, Zanolini A, Mulenga L, Sikazwe I (2015). Elevated AST-to-platelet ratio index is associated with increased all-cause mortality among HIV-infected adults in Zambia. Liver Int..

[74] Halfon P, Bacq Y, De Muret A, Penaranda G, Bourliere M, Ouzan D (2007). Comparison of test performance profile for blood tests of liver fibrosis in chronic hepatitis C. J Hepatol..

[75] Passos AM, Treitinger A, Spada C (2010). An overview of the mechanisms of HIV-related thrombocytopenia. Acta Haematol..

[76] Hull MW, Rollet K, Odueyungbo A, Saeed S, Potter M, Cox J (2012). Factors associated with discordance between absolute CD4 cell count and CD4 cell percentage in patients coinfected with HIV and hepatitis C virus. Clin Infect Dis..

[77] Price JC, Seaberg EC, Badri S, Witt MD, D'Acunto K, Thio CL (2012). HIV monoinfection is associated with increased aspartate aminotransferase-to-platelet ratio index, a surrogate marker for hepatic fibrosis. J Infect Dis..

[78] Mendeni M, Foca E, Gotti D, Ladisa N, Angarano G, Albini L (2011). Evaluation of liver fibrosis: concordance analysis between noninvasive scores (APRI and FIB-4) evolution and predictors in a cohort of HIV-infected patients without hepatitis C and B infection. Clin Infect Dis..

[79] Singal AG, Thomassen LV, Gretch DR, Shuhart MC (2011). Use of the AST to platelet ratio index in HCV/HIV co-infected patients. Aliment Pharmacol Ther..

[80] Macias J, Giron-Gonzalez JA, Gonzalez-Serrano M, Merino D, Cano P, Mira JA (2006). Prediction of liver fibrosis in human immunodeficiency virus/hepatitis C virus coinfected patients by simple non-invasive indexes. Gut..

[81] Smith JO, Sterling RK (2009). Systematic review: non-invasive methods of fibrosis analysis in chronic hepatitis C. Aliment Pharmacol Ther..

[82] Blau N, Duran M, Gibson K (2008). Laboratory Guide to the Methods in Biochemical Genetics.

[83] Salbach J, Rachner TD, Rauner M, Hempel U, Anderegg U, Franz S (2012). Regenerative potential of glycosaminoglycans for skin and bone. J Molecul Med (Berlin, Germany)..

[84] Gressner OA, Weiskirchen R, Gressner AM (2007). Biomarkers of liver fibrosis: clinical translation of molecular pathogenesis or based on liver-dependent malfunction tests. Int J Clin Chem..

[85] Montazeri G, Estakhri A, Mohamadnejad M, Nouri N, Montazeri F, Mohammadkani A (2005). Serum hyaluronate as a non-invasive marker of hepatic fibrosis and inflammation in HBeAg-negative chronic hepatitis B. BMC Gastroenterol..

[86] Parsian H, Rahimipour A, Nouri M, Somi MH, Qujeq D (2010). Assessment of liver fibrosis development in chronic hepatitis B patients by serum hyaluronic acid and laminin levels. Acta Clin Croatica..

[87] Zhang YX, Wu WJ, Zhang YZ, Feng YL, Zhou XX, Pan Q (2008). Noninvasive assessment of liver fibrosis with combined serum aminotransferase/platelet ratio index and hyaluronic acid in patients with chronic hepatitis B. World J Gastroenterol..

[88] Arain SA, Meo SA, Jamal Q (2011). Serum hyaluronic acid level does not reliably differentiate minimal and significant liver disease in chronic hepatitis C. Saudi Med J..

[89] Patel K, Lajoie A, Heaton S, Pianko S, Behling CA, Bylund D (2003). Clinical use of hyaluronic acid as a predictor of fibrosis change in hepatitis C. J Gastroenterol Hepatol..

[90] Peters L, Mocroft A, Soriano V, Rockstroh J, Rauch A, Karlsson A (2013). Hyaluronic acid levels predict risk of hepatic encephalopathy and liver-related death in HIV/viral hepatitis coinfected patients. PLoS One..

[91] Rostami S, Parsian H (2013). Hyaluronic Acid: from biochemical characteristics to its clinical translation in assessment of liver fibrosis. Hepatitis Monthly..

[92] Hakim ST, Alsayari M, McLean DC, Saleem S, Addanki KC, Aggarwal M (2008). A large number of the human microRNAs target lentiviruses, retroviruses, and endogenous retroviruses. Biochem Biophysic Res Commun..

[93] Houzet L, Jeang KT (2011). MicroRNAs and human retroviruses. Biochim Biophysic Acta..

[94] Egana-Gorrono L, Escriba T, Boulanger N, Guardo AC, Leon A, Bargallo ME (2014). Differential microRNA expression profile between stimulated PBMCs from HIV-1 infected elite controllers and viremic progressors. PLoS One..

[95] Skalsky RL, Cullen BR (2010). Viruses, microRNAs, and host interactions. Annual Rev Microbiol..

[96] Jopling CL, Yi M, Lancaster AM, Lemon SM, Sarnow P (2005). Modulation of hepatitis C virus RNA abundance by a liver-specific MicroRNA. Science (New York, NY..

[97] Zhu Z, Wilson AT, Mathahs MM, Wen F, Brown KE, Luxon BA (2008). Heme oxygenase-1 suppresses hepatitis C virus replication and increases resistance of hepatocytes to oxidant injury. Hepatology (Baltimore, Md..

[98] Bihrer V, Waidmann O, Friedrich-Rust M, Forestier N, Susser S, Haupenthal J (2011). Serum microRNA-21 as marker for necroinflammation in hepatitis C patients with and without hepatocellular carcinoma. PLoS One..

[99] Chen Y, Chen J, Wang H, Shi J, Wu K, Liu S (2013). HCV-induced miR-21 contributes to evasion of host immune system by targeting MyD88 and IRAK1. PLoS Pathog..

[100] Zeitels LR, Acharya A, Shi G, Chivukula D, Chivukula RR, Anandam JL (2014). Tumor suppression by miR-26 overrides potential oncogenic activity in intestinal tumorigenesis. Gen Dev..

[101] Jangra RK, Yi M, Lemon SM (2010). Regulation of hepatitis C virus translation and infectious virus production by the microRNA miR-122. J Virol..

[102] Xing TJ, Jiang DF, Huang JX, Xu ZL (2014). Expression and clinical significance of miR-122 and miR-29 in hepatitis B virus-related liver disease. Genet Mol Res..

[103] Cheng HR, Kao JH, Wu HL, Tseng TC, Liu CH, Yang HC (2015). Clinical significance of circulating miR-122 in patients with dual chronic hepatitis B and C virus infection. Hepatol Int..

[104] Song K, Han C, Dash S, Balart LA, Wu T (2015). MiR-122 in hepatitis B virus and hepatitis C virus dual infection. World J Hepatol..

[105] Gupta A, Swaminathan G, Martin-Garcia J, Navas-Martin S (2012). MicroRNAs, hepatitis C virus, and HCV/HIV-1 co-infection: new insights in pathogenesis and therapy. Viruses..

[106] Triboulet R, Mari B, Lin YL, Chable-Bessia C, Bennasser Y, Lebrigand K (2007). Suppression of microRNA-silencing pathway by HIV-1 during virus replication. Science (New York, NY..

[107] Manfe V, Biskup E, Rosbjerg A, Kamstrup M, Skov AG, Lerche CM (2012). miR-122 regulates p53/Akt signalling and the chemotherapy-induced apoptosis in cutaneous T-cell lymphoma. PLoS One..

[108] Bandyopadhyay S, Friedman RC, Marquez RT, Keck K, Kong B, Icardi MS (2011). Hepatitis C virus infection and hepatic stellate cell activation downregulate miR-29: miR-29 overexpression reduces hepatitis C viral abundance in culture. J Infect Dis..

[109] Nathans R, Chu CY, Serquina AK, Lu CC, Cao H, Rana TM (2009). Cellular microRNA and P bodies modulate host-HIV-1 interactions. Molecul cell..

[110] Wang X, Ye L, Hou W, Zhou Y, Wang YJ, Metzger DS (2009). Cellular microRNA expression correlates with susceptibility of monocytes/macrophages to HIV-1 infection. Blood..

[111] Xu J, Wu C, Che X, Wang L, Yu D, Zhang T (2011). Circulating microRNAs, miR-21, miR-122, and miR-223, in patients with hepatocellular carcinoma or chronic hepatitis. Molecul Carcinogen..

[112] Witwer KW, Watson AK, Blankson JN, Clements JE (2012). Relationships of PBMC microRNA expression, plasma viral load, and CD4+ T-cell count in HIV-1-infected elite suppressors and viremic patients. Retrovirology..

[113] Anadol E, Schierwagen R, Elfimova N, Tack K, Schwarze-Zander C, Eischeid H (2015). Circulating microRNAs as a marker for liver injury in human immunodeficiency virus patients. Hepatology (Baltimore, Md..

